# A rare case of gastro-intestinal stromal tumor presented with hematemesis and severe anemia from a low and middle-income country

**DOI:** 10.1016/j.ijscr.2024.109456

**Published:** 2024-03-04

**Authors:** Md. Saiful Islam, Abhigan Babu Shrestha, Fahmida Rimti, Suju Bhattarai, Md. Raihan Kabir Ziko, Barsha Pantha

**Affiliations:** a Department of Surgery, M Abdur Rahim Medical College hospital, Dinajpur, Bangladesh; b Department of Internal Medicine, M Abdur Rahim Medical College hospital, Dinajpur, Bangladesh; c Chittagong Medical College, Chattogram 4203, Bangladesh; d Kathmandu Medical College and Teaching Hospital, Kathmandu, Nepal; e Department of Surgery, Post Graduation Institute of Medical Education and Research, Chandigarh, India

**Keywords:** Case reports, Hematemesis, Mortality, GIST

## Abstract

**Introduction and importance:**

Gastrointestinal stromal tumors (GISTs) are rare mesenchymal tumors with varied clinical presentations. This case report highlights the significance of recognizing atypical GIST presentations, such as hematemesis, especially in resource-limited settings.

**Case presentation:**

A 52-year-old male from an economically disadvantaged background presented with hematemesis and severe anemia. Initial investigations suggested iron deficiency anemia, but further evaluation revealed a gastric mass, raising suspicion of GIST. Despite limited resources, a distal radical gastrectomy confirmed the GIST diagnosis, and the patient underwent surgical resection followed by imatinib therapy.

**Clinical discussion:**

This case underscores the diagnostic challenges posed by GISTs and the importance of imaging studies, given their often nonspecific symptoms. Limited resources and economic constraints in low-income settings can hinder comprehensive diagnosis and treatment. Access to specialized oncological services is crucial for accurate management.

**Conclusion:**

Early recognition and management of GIST, even in atypical presentations like hematemesis, can significantly impact patient outcomes. This case report highlights the need for improved healthcare infrastructure in low-resource settings and calls for initiatives to ensure equitable access to diagnostic tests and appropriate treatment for rare diseases like GIST.

## Highlights

• This case presents a 52-year-old male with hematemesis as a manifestation of GIST
• Diagnostic workup for GIST includes ebdoscopy with biopsy, ultrasound and CT scan, Immunohistochemistry testing is crucial for definitive diagnosis.
• Surgical resection is the mainstay of treatment for GIST, and targeted therapies such as imatinib may be used to control tumor growth.

## 1 Introduction

Gastrointestinal stromal tumors (GIST) are rare mesenchymal tumors originating in the gastrointestinal tract, anywhere from the esophagus to the anus [1]. Currently, only 3 % of gastrointestinal (GI) tumors are GIST [2]. Depending on the size, location, proximity to the gastrointestinal wall, and malignant potential of the GIST, symptoms may vary. Small tumors, however, could be asymptomatic or cause vague gastrointestinal symptoms, while some may manifest slowly due to their long latency period. However, hematemesis is one of its vague presentations.

Various imaging modalities like Ultrsonogram of the abdomen, CT scan and endoscopy combined with biopsy are used to diagnose GIST [3]. The main course of treatment entails surgical removal of the tumor, but other treatments, including imatinib or other targeted medications, may be used to reduce or control the tumor's growth [4].

We present a 52 years old man with hematemesis due to a GIST of the stomach. This case is written in line with SCARE and PROCESS guidelines [5,6].

## 2 Case presentation

A 52-year-old male presented at our hospital's emergency department with complaints of hematemesis and fatigue persisting for 8 months. He is normotensive, non-diabetic, and comes from an economically disadvantaged family. No other family members have reported similar issues.

Previously, the patient was in reasonable health until 8 months ago when he began experiencing nonprojectile hematemesis in small amounts. He also acknowledged having black, tarry stools with no accompanying abdominal pain. Following these symptoms, he gradually developed fatigue and shortness of breath. These symptoms progressed over time and were not associated with body position, seasonal changes, allergies, or diurnal variation.

During the physical examination, severe anemia was observed, along with a blood pressure reading of 100/70 mmHg and mild dehydration. Abdominal examination did not reveal any significant abnormalities, but a cardiac examination indicated the presence of a murmur due to anemia on the left sternal border.

Routine investigations revealed a hemoglobin (Hb) level of 4.7 g/dl, along with other blood profiles as shown in Table 1. The red cell values indicated microcytic hypochromic anemia, suggesting a possible case of iron deficiency anemia. This was further confirmed by a peripheral blood film (PBF) showing numerous pencil cells, as well as a serum ferritin level of 12.3 ng/ml (normal range: 20–300 ng/ml).

**Table 1:** Laboratory report of blood findings for anemia.

Parameter	Range	Normal Range
RBC	2.99 m/ul	4.5–6.5,/ul
HCT/PCV	17.9 %	40–54 %
MCV	59.7 fL	76–94 fL
MCH	15.8 pg	27–32 pg
MCHC	26.4 g/dl	29–34 g/dl
RDW-cv	16.9 %	10–16 %
ESR	117	0–10 mm/1st hr

**Fig. 1:**
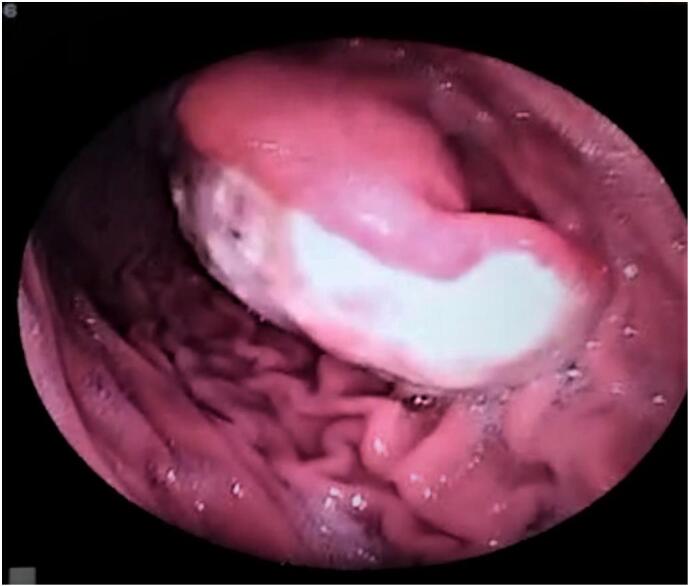
Upper GI endoscopy revealing ulcerated mass in the stomach.

An abdominal ultrasound revealed a gastric growth measuring 6.43 *cm, and an upper gastrointestinal endoscopy showed an ulcerated mass on the gastric antrum measuring 8.6* cm (see Fig. 1). A biopsy was taken from the mass, which revealed inflamed and congested mucosa with ulceration, inflammatory exudate, granulation tissue formation, and regenerative changes. As the tumor was located beneath the mucosa, a definitive diagnosis of gastrointestinal stromal tumor (GIST) could not be established. The patient was advised to undergo a CT scan of the abdomen for further evaluation, but due to financial constraints, he declined the procedure. Other routine investigations yielded results within normal parameters.

Initially, conservative management was implemented during the patient's admission, and his anemia was corrected through the administration of 5 units of packed red blood cells on consecutive days, along with calcium gluconate injections. After correcting the anemia, given the patient's condition and financial circumstances, our hospital offered to cover the expenses of a routine operation. A sub total gastrectomy was performed under general anesthesia, and a specimen was sent for histopathological examination.

The histopathology report revealed a malignant tumor consisting of spindle-shaped cells with hyperchromatic nuclei arranged diffusely. Several atypical mitoses were observed at a rate of 3–5 per high-power field. Lymph nodes displayed reactive changes. These findings were consistent with a diagnosis of GIST. Further immunohistochemistry evaluations (CD 117 and CD 34) were recommended for a comprehensive assessment; however, these tests were not available at our hospital.

The patient remained hospitalized until the 7th postoperative day and was subsequently discharged with a prescription for Imatinib 400 mg once daily. He was advised to seek further evaluation and management from an oncologist for his condition. Follow up was advised as per the surgeon and oncologist.

## 3 Discussion

Gastrointestinal stromal tumors (GISTs) have a genetic basis and resemble other hereditary oncologic syndromes like VHL, MEN 1 and 2, and the Carney complex. This similarity in pathophysiology can sometimes obscure the diagnosis or lead to confusion with other mesenchymal neoplasms of the gastrointestinal tract, such as leiomyoma or leiomyosarcoma. To obtain a precise diagnosis, the utilization of immunohistochemical resources is imperative. These resources encompass markers such as KIT, CD117, CD34, S-100 protein, Actin, and Desmin. Furthermore, complementary imaging studies are essential in the diagnostic process [7].

Although GISTs are the most common benign non-epithelial neoplasms of the gastrointestinal tract, they still account for only 1 % of all primary tumors in this region [8]. The worldwide incidence of GISTs is estimated to be between 11 and 19.6 cases per million population, with variations in different regions. Unfortunately, the specific incidence and prevalence of GISTs in Bangladesh are unknown. However, recent studies in the United States have reported an annual incidence ranging from 4000 to 6000 new cases, corresponding to approximately 7 to 20 cases per 1,000,000 population per year [[8], [9], [10]].

Diagnosing GISTs is challenging due to their often silent or nonspecific abdominal symptoms. However, the growing utilization of CT scans in abdominal investigations has led to a rise in identifying incidental GISTs. Approximately 25 % of cases manifest with recurrent bleeding, causing melena, hematemesis, and anemia [11]. Additional indications include early satiety, abdominal pain, palpable mass, and potential surgical emergencies like bowel obstruction, perforation, or gastrointestinal hemorrhage [12].

In this case report, we describe the unusual presentation of GIST as hematemesis in a 52-year-old male from a low-income background. The patient's chief complaints of hematemesis and fatigue raised suspicion of a gastrointestinal lesion. It indicates upper gastrointestinal bleeding from various etiologies such as peptic ulcers, varices, or tumors 13]. In this case, the patient's history of black, tarry stools, fatigue, and severe anemia indicated chronic bleeding from the gastrointestinal tract.

The initial diagnostic workup revealed iron deficiency anemia, a common finding in gastrointestinal bleeding [11]. The anemia was severe, with a hemoglobin level of 4.7 g/dl, and required immediate correction to stabilize the patient's condition. The subsequent imaging studies, including abdominal ultrasound and upper gastrointestinal endoscopy and biopsy, provide additional information about the cause of hematemesis. However, the initial biopsy taken from the mass did not establish the diagnosis of GIST, as it only showed inflamed and congested mucosa with ulceration and inflammatory exudate.

The initial endoscopic biopsy, although inconclusive, demonstrated inflamed and congested mucosa with ulceration and inflammatory exudate. The presence of granulation tissue and regenerative changes further indicated a pathological process. However, the underlying GIST was not identified at this stage, highlighting the challenges in diagnosing GISTs based on endoscopic biopsies alone.

Imaging studies, such as ultrasonography and computed tomography (CT), play a crucial role in the diagnosis and staging of GISTs [14]. In this case, ultrasonography revealed a gastric mass, while upper gastrointestinal endoscopy identified an ulcerated mass in the gastric antrum. These findings raised suspicion about GIST and prompted further evaluation.

Per operative findings, a mobile gastric mass was localized within the distal stomach that didn't invade the serous layer. There was no locoregional invasion and intra-abdominal lymphadenopathy or ascites. So, we did Distal radical gastrectomy and the histopathological examination of the resected specimen confirmed the presence of GIST in the stomach. The characteristic features of GIST include spindle-shaped cells with hyperchromatic nuclei and the presence of atypical mitotic figures. Unfortunately, due to limited resources, immunohistochemistry testing for CD117 and CD34, which are essential for definitive diagnosis and risk stratification of GIST [7], was not available in the hospital.

The definitive diagnosis of GIST was made based on the histopathological examination of the resected specimen obtained during distal radical gastrectomy. The presence of spindle-shaped cells with hyperchromatic nuclei, along with the identification of 3–5 atypical mitotic figures per high-power field, supported the diagnosis of GIST. Unfortunately, due to limited resources, immunohistochemistry for further evaluation, including the assessment of CD117 and CD34 expression, was not available in our hospital.

Management of GIST typically involves surgical resection [15], and in this case, the patient underwent satisfactory distal radical gastrectomy & Roux en Y gastrojejunostomy using three raw GI surgical staplers. There was no visible residual tumor. The decision for surgical intervention was based on the size of the tumor, its ulcerated appearance, excessive & recurrent hematemesis and the patient's overall condition. The postoperative period was uneventful. Following surgery, the patient was started on imatinib therapy, a tyrosine kinase inhibitor that targets the KIT receptor, which is commonly expressed in GISTs. Imatinib therapy has been shown to improve outcomes in advanced or metastatic GIST.

The challenges faced in this case due to limited resources and the patient's economic constraints highlight the disparities in healthcare access and the need for improved healthcare infrastructure in low-resource settings. The lack of availability of certain diagnostic tests, such as immunohistochemistry, limited our ability to characterize the tumor and tailor the treatment accordingly fully. Access to comprehensive diagnostic facilities and specialized oncological services is crucial for accurate diagnosis and appropriate management of rare diseases like GIST.

This case report underscores the importance of early recognition and management of GIST, particularly in atypical presentations, such as hematemesis. Timely diagnosis and intervention can significantly impact patient outcomes. Furthermore, efforts should be directed toward improving healthcare infrastructure and resource availability in low-resource settings to ensure equitable access to diagnostic tests and appropriate treatment modalities.

## 4 Conclusion

This case report highlights a rare presentation of GIST as hematemesis in a patient from a low-income background. This case emphasizes the importance of early recognition, accurate diagnosis, and appropriate management of GIST in resource-limited settings. Further research and initiatives are warranted to improve healthcare accessibility and ensure optimal care for patients with rare diseases like GIST.

## Declaration of patient consent

Written informed consent was obtained from the patient for publication of this case report and accompanying images. A copy of the written consent is available for review by the Editor-in-Chief of this journal on request.

## Ethical approval

This case report adheres to the principles outlines in the declaration of Helsinki snd its subsequent revisions. The patient’s informed consent was obtained prior to their inclusion of their data in the case report, ensuring confidentiality and privacy. As it is a case report involving individual patient, it does not require formal ethical approval by and IRB committee as this does not involve any interventions or experimental procedures. It was waived by M Abdur Rahim Medical College hospital, Dinajpur, Bangladesh.

## Funding

N/A

## Author contribution

Md. Saiful Islam: Conceptualization, Visualization, Patient management and care

Abhigan Babu Shrestha, Fahmida Rimti, Suju Bhattarai, Md. Raihan Kabir Ziko, Barsha Panta all contributed equally

## Guarantor

Fahmida Hoque Rimti

rimti.001@gmail.com

## Declaration of competing interest

The authors declare no conflicts of interest.

## Data Availability

Data available on request from the authors.
